# Editorial: Public health approaches to ending violence against women: global campaigns, policy, and community action

**DOI:** 10.3389/fpubh.2025.1770777

**Published:** 2026-01-13

**Authors:** Franklin N. Glozah, Amira Shaheen

**Affiliations:** 1 Department of Social and Behavioural Sciences, School of Public Health, University of Ghana, Accra, Ghana; 2 Division of Public Health, An-Najah National University, Nablus, Palestine

**Keywords:** community intervention, gender response, intimate abusive relationship, public health, violence—prevention and control

Violence against women continues to be one of the most prevalent public health challenges of our time, affecting the safety, wellbeing and life opportunities of women and girls across every region of the world. Its repercussions extend far beyond immediate physical harm, influencing emotional health, family stability, social participation and long-term economic security. Although governments and communities have made commitments toward ending violence, the drivers of abuse remain deeply embedded in social norms, gendered power relations, and structural inequalities. This Research Topic brings together seven studies that collectively deepen understanding of the burden of violence, the factors that sustain it and the approaches needed to prevent harm and promote recovery. In doing so, the Research Topic provides a clear, evidence based roadmap for pubic health practitioners seeking practical and scalable solutions.

Two contributions examine the health consequences and epidemiological patterns of violence. One study aimed to investigate how experiences of domestic abuse influence anxiety, depression and overall wellbeing among women in Saudi Arabia, providing evidence of the lasting emotional and psychological toll associated with violence. The findings highlight the urgent need to strengthen mental health support, integrate psychosocial care into routine services for survivors and ensure that health systems can respond sensitively and effectively (Al Shahrani and Hammad). A second study sought to analyse how experiences of violence in China vary across age groups, time periods and generational cohorts. Using age–period–cohort modeling, the authors show how shifting social expectations, cultural patterns and historical contexts influence the vulnerability of different groups of women. Such clarity in identifying temporal and generational differences underscores the importance of tailoring prevention strategies rather than adopting one-size-fits- all approaches. This work reinforce the importance of generationally attuned strategies that respond to broader social change (Shen and Xie).

Preventing violence also requires close attention to the social and cultural environments in which it takes root. A multilevel analysis from East Africa aimed to examine how men's approval of physical intimate partner violence is shaped by both individual attitudes and community norms. The study demonstrates that harmful beliefs are reinforced within broader social environments, where gender expectations, community approval and entrenched power relations legitimize violence and undermine efforts toward equality. These findings make clear that prevention efforts must extend beyond individual-level interventions to address the collective norms and structures that perpetuate harm, and must actively involve men and boys as key partners in promoting respectful, non-violent relationships (Demissie et al.). Engaging men in this work is not optional; it is central to dismantling the very norms that sustain violence.

Two articles focus on strengthening interventions and building capacity within communities and health systems. One study aimed to evaluate the effectiveness of couple-focused educational sessions delivered in rural Ethiopia, designed to improve communication, foster mutual respect and reduce controlling behavior. The intervention led to improvements in knowledge, attitudes and relationship dynamics, alongside reductions in behavioral indicators associated with violence. These results illustrate the potential of locally grounded, culturally relevant programmes to transform interpersonal relationships and reduce the risk of violence at community level. They also highlight the value of working directly with couples to challenge harmful norms and build skills that promote safety and wellbeing (Chen et al.). A complementary study from China investigated the readiness of nursing students to identify, support and refer survivors of intimate partner violence. While raising awareness among students represents progress, the study identifies persistent gaps in training, institutional systems, and confidence in responding appropriately. The findings point to a clear need for structured curricular reform, standardized competencies and sustained institutional support to ensure that future practitioners are adequately prepared.

A structural perspective is provided by an analysis of gender responsive budgeting in India during the post-pandemic period. This study examines how national budgeting processes, public finance mechanisms and policy priorities influence gender equality and national responses to violence. The authors show that meaningful progress depends on sustained government commitment, adequate resource allocation and transparent accountability mechanisms. Their work reinforces the idea that policy frameworks, fiscal planning and national budgets are powerful tools for shaping the conditions in which women live and for supporting long-term strategies aimed at reducing violence. In this sense, gender responsive budgeting is itself a public health intervention, capable of influencing the environments that either protect or endanger women (Barman and Gupta). This study also reinforce that without political will and financial commitment, policy aspirations remain symbolic rather than actionable.

A final contribution presents a global assessment of depressive disorders among women of childbearing age over three decades, with the aim of examining how mental health risks evolve across time and contexts. Although not limited to survivors of violence, the study highlights the broader vulnerabilities experienced by women and shows how socio-economic pressures, caregiving responsibilities and gendered expectations intersect to influence mental health outcomes. By situating violence within a wider landscape of structural disadvantage, the study underscores the need for integrated policies that address both direct and indirect determinants of women's health. This work serves as an important reminder that violence does not occur in isolation but interacts with other forms of disadvantage and inequality, shaping the broader health landscape for women across the life course (Yang et al.).

Collectively, these seven studies underscore that violence against women cannot be understood as a single event or isolated health issue. Rather, it emerges through an interplay of individual behaviors, community norms, economic structures, institutional capacities and national policy environments. Effective prevention and response therefore require coordinated action across multiple levels. At the individual and family level, supportive, respectful and equitable relationships must be nurtured. At community level, harmful norms must be challenged, and advocacy efforts must be grounded in local realities. Health systems must be equipped to recognize signs early, provide compassionate care and link survivors to support networks. At the structural level, gender responsive financing and policy reform ensure that commitments to ending violence are supported by long-term investment and political will. Only through unified, multi-sectoral engagement can sustainable progress be achieved.

Future research will play an important role in expanding this evidence base. Emerging issues such as digital forms of violence, coercive control in online spaces and technology-facilitated harassment require further attention. There is also a need for more work on intersecting vulnerabilities linked to disability, migration, socioeconomic hardship and other factors that compound risk. Further research is also urgently needed to understand the heightened patterns of GBV in conflict and war settings, including the use of sexual violence as a weapon against women, and to document how military actions intensify risks and undermine access to protection and services. In addition, evaluating and scaling community-based interventions remain essential for ensuring that promising models across diverse contexts. Robust monitoring and evaluation frameworks will be indispensable to track progress, ensure accountability and guide evidence-informed action.

This Research Topic illustrates the depth and breadth of public health approaches required to address violence against women. The contributions highlight the importance of combining community action, health system strengthening and structural reform, and reaffirm that ending violence demands persistent, coordinated and evidence-informed efforts. Taken together, these studies offer practitioners and policymakers a compelling, action-oriented body of evidence to guide future strategies aimed at building safer environments for women and girls.

